# Synapto-protective effect of lithium on HIV-1 Tat-induced synapse loss in rat hippocampal cultures

**DOI:** 10.1080/19768354.2021.2018044

**Published:** 2021-12-27

**Authors:** Namgue Hong, Jeong-Soo Park, Hee Jung Kim

**Affiliations:** a Department of Physiology, College of Medicine, Dankook University, Cheonan, Republic of Korea; b Department of Medical Laser, Graduate School, Dankook University, Cheonan, Republic of Korea; c Medical Laser Research Center, College of Medicine, Dankook University, Cheonan, Republic of Korea; d Department of Biochemistry, College of Medicine, Dankook University, Cheonan, Republic of Korea

**Keywords:** Lithium, HIV-1 Tat, PSD95, synapse loss, neuronal cell death

## Abstract

Human immunodeficiency virus type I (HIV-1) infection of the CNS produces synapse loss which correlates with cognitive decline in patients with HIV-associated neurocognitive disorders (HAND). Lithium is mood stabilizer of unknown mechanism used to treat bipolar disorder and is known to exhibit neuroprotective properties. Here, we studied the effects of lithium on HIV-1 Tat-induced synapses between rat hippocampal neurons. The number of synapses was quantified to detect clusters of the scaffold protein postsynaptic density 95 (PSD95) which is clustered at glutamatergic synapses on cultured rat hippocampal neurons *in vitro*. Lithium protected synapses from HIV-1 Tat-induced synapse loss and subsequent neuronal death. This synaptic protection was prevented by both the activation of NMDA receptor leading to intracellular signaling and the regulatory pathway of lithium including inositol depletion and glycogen synthase kinase-3*β* (GSK-3*β*). These results suggest that mood stabilizers might be effective drugs to treat neurodegenerative disorders including HAND.

## Introduction

About 30 million people worldwide are infected with human immunodeficiency virus-1 (HIV-1), and not all HIV infected patients progress to AIDS (Kaul et al. 2001). HIV-associated neurocognitive disorders (HAND) afflict approximately 30–50% of HIV-infected patients (Tozzi et al. 2005). In the CNS, HIV-1 infects astrocytes, macrophages, and microglia, but not neurons. Infected cells release neurotoxic factors such as inflammatory cytokines and viral proteins (Genis et al. 1992). HIV transactivator of transcription (Tat) is a toxic viral protein released from HIV-1 infected cells (King et al. 2006). HIV-1 Tat mRNA is elevated in the CNS of HAND patients (Joshi et al. 2020). Tat protein produces HAND neuropathologies (Kim et al. 2003; Fitting et al. 2010) and impaired cognitive function (Raybuck et al. 2017). HIV-1 Tat also elicits dendritic pruning, decreased spine density and synapse loss (Eugenin et al. 2007; Kim et al. 2008). Dendritic damage and synapse loss correlate with cognitive decline in HAND patients (Sa et al. 2004).

HIV-1 Tat causes excitatory synaptic damage via a mechanism distinct from that leading to cell death (Kim et al. 2008). Tat binds to low-density lipoprotein receptor-related protein (LRP) forming a macromolecular complex including LRP, postsynaptic density protein-95 (PSD-95), *N*-methyl-d-aspartic acid (NMDA)_receptors, and nNOS (Eugenin et al. 2007) resulting in the potentiation of NMDA receptors (Krogh et al. 2015), increasing calcium influx and ultimately inducing the loss of postsynaptic density via calcium-induced activation of an ubiquitin ligase (Bonavia et al. 2001; Kim et al. 2008). This calcium influx induces cell death at the same time via activating neuronal nitric oxide synthase (nNOS) pathway.

Previous studies reported that many patients with HIV infection has suffered from mood disorders, especially depression and manic syndrome (Angelino and Treisman 2001; Arseniou et al. 2014). HIV mainly affects subcortical brain areas (Woods et al. 2009) known to control mood (Hibar et al. 2015). Therefore, it is, intriguing to note that there is correlation between HIV-induced cognitive decline and mood disorders (Rubin and Maki 2019). Many mood stabilizers have been used to treat bipolar disorder, with, lithium being the most widely used mood stabilizer for decades (Machado-Vieira et al. 2009; Chiu et al. 2013).

Lithium can protect neurons in many neuronal injury models (Dell’Osso et al. 2016). Stabilization of neuronal Na^+^/K^+^ ion channels, reduction of neuronal inflammation, and regulation of anti- and pro-apoptotic protein expression might be involved in the neuroprotective mechanism of lithium (Lowthert et al. 2012; Beurel and Jope 2014; Dwivedi and Zhang 2014; Nassar and Azab 2014; Jakobsson et al. 2017; Qaswal 2020). Lithium affords protection from murine immunodeficiency (Gallicchio et al. 1993; Dou et al. 2005). It can also have neuroprotective effects to patients with HIV-associated dementia (HAD) (Harvey et al. 2002). In addition, lithium can prevent gp120-induced neurodegeneration (Everall et al. 2002) through inhibition of GSK-3β (Manji et al. 1999). The protective action of lithium against HIV-1 Tat-induced neurotoxicity has also been shown (Maggirwar et al. 1999). In clinical trials, individuals with symptomatic HIV-associated neurocognitive impairment treated with lithium have also shown neuropsychological improvements (Letendre et al. 2006). The effect of lithium on the synaptic dysfunction caused by HIV-1 Tat, and which precedes neuronal death has not been reported yet.

The objective of this study was to determine the role of lithium as a therapeutic agent in HIV-1 Tat-induced synapse loss and the mechanism involved in the regulation of synapse loss by lithium. Here we show that lithium prevented synaptic damage and subsequent death on cultured rat hippocampal neurons caused by HIV-1 Tat. The synaptic protection produced by lithium appears to be mediated by both GSK-3β inhibition and inositol depletion. These results provide mechanistic insight into the potential of using mood stabilizers to treat synaptic dysfunction caused by HIV-1.

## Materials and methods

### Antibodies and chemicals

The antibodies used and their manufacturers are as follows. PSD95 (51-6900) was purchased from Invitrogen. MAP2 (M9942) was purchased from Sigma-Aldrich. The following chemicals were used: HIV-1 Tat Clade-B (ProSpec, HIV-129), lithium chloride (Sigma-Aldrich, L9650), valproic acid (Sigma-Aldrich, P4543), L-NAME (Sigma-Aldrich, N5751), nutlin-3 (Sigma-Aldrich, N6287), AP5 (Tocris, 0106), PP2 (Tocris, 1407), PP3 (Tocris, 2794), myo-Inositol (Sigma-Aldrich, I7508) and SB216763 (Sigma-Aldrich, S3442).

### Cell culture

Rat hippocampal neurons were grown in primary culture as described previously (Kim et al. 2008) with some modifications. Briefly, fetuses were removed from maternal rats anesthetized with 16.5% urethane on embryonic day 17. Hippocampi were dissected and placed in Ca^2+^- and Mg^2+^-free HEPES-buffered Hanks’ salt solution (HHSS, pH 7.45, 20 mM HEPES, 137 mM NaCl, 1.3 mM CaCl_2_, 0.4 mM MgSO_4_, 0.5 mM MgCl_2_, 5.0 mM KCl, 0.4 mM KH_2_PO_4_, 0.6 mM Na_2_HPO_4_, 3.0 mM NaHCO_3_, and 5.6 mM glucose). Cells were dissociated by trituration using a 5-ml pipette and a flame-narrowed Pasteur pipette. Cells were then, pelleted and resuspended in neurobasal medium without L-glutamine with 2% B27 supplement, 0.25% Glutamax I and penicillin/streptomycin/amphotericin B (100 U/ml, 100 and 0.025 µg/mL, respectively). Dissociated cells were then plated onto 25-mm round cover glass at a density of 80,000 cells/well. The cover glass was pre-coated with Matrigel (0.2 mg/ml; BD Bioscience). Neurons were grown in a humidified atmosphere at 37°C with 10% CO_2_ and 90% air, at pH 7.4. They were fed on days 3, 7, and 10 by replacing 75% of spent media with fresh media. Each experiment was performed in at least three independent cultures.

### Immunocytochemistry

Hippocampal cultures were prepared as described above and fixed on day 12 *in vitro*. Cells were washed with PBS, and fixed with ice-cold methanol for 10 min at –20°C. Cells were washed with PBS three times and blocked with 10% bovine serum albumin (BSA; Sigma-Aldrich) in PBS for 1 h at room temperature. After blocking, cells were incubated with affinity purified antibodies in PBS for 16 h at 4°C. Mouse anti-MAP2 (Sigma-Aldrich) and rabbit anti-PSD95 (Abcam) antibodies were used to identify neurons and synapses, respectively. Immuno-labeled cells were visualized after incubating with Alexa Fluor 488 anti-rabbit IgG (Invitrogen) and Alexa Fluor 555 anti-mouse IgG (Invitrogen) in PBS for 1 hr at room temperature. After washing in phosphate-buffered saline, coverslips were inverted on slides over a drop of VECTASHIELD Mounting Medium (Vector Laboratories, Inc., Burlingame, CA, USA). Neurons labeled with Alexa Fluor 488 (excitation, 488 nm; emission, >519 nm) and Alexa Fluor 555 (excitation, 555 nm; emission, >565 nm) were observed under a confocal microscope.

### MTT assay

To determine cell viability, cortical neurons were treated neurobasal medium with HIV-1 Tat (50 ng/ml) supplemented with 2% B27 or neurobasal medium only with 2% B27 supplement as a control for 24, 48 and 72 h. Cells were incubated with 3-[4,5-dimethylthiazol-2-yl]-2,5-dipheyltetrazolium bromide (MTT) at a final concentration of 0.5 mg/ml for 4 h (Choi et al. 2021). Dark blue formazan crystals formed in intact cells were dissolved in dimethyl sulfoxide and the absorbance value of each well was measured at wavelength of 570 nm with a microplate reader.

### Confocal imaging

Neurons were transferred to the stage of a laser-scanning confocal microscope (LSM 700, Carl Zeiss, Germany) and viewed with a 40× objective (numerical aperture, 1.3). Eight optical sections spanning 8 µm in *z*-dimension were collected (1-µm steps) and combined through *z*-axis into a compressed *z* stack (Woo et al. 2020; Kim et al. 2021). PSD95 was excited at 488 nm with an argon ion laser. Emission was collected at 519 nm (10 nm bandpass). The excitation (HeNe laser) and emission wavelengths for MAP2 were 555 nm and >565 nm, respectively.

### Image processing

Images were obtained with identical acquisition parameters for all conditions in a particular experiment. They were analyzed using ZEN 2010 software (Carl Zeiss). PSD95 was quantified by thresholding fluorescence intensity of GFP in ZEN 2010 to define outlines of neurons. Intensity values were calculated and normalized to the average fluorescence intensity of untreated cells. The number of synaptic sites labeled by PSD95 derived from a single neuron recognized as MAP2 was also quantified using the image processing algorithm previously described (Kim et al. 2021). At least three neurons from randomly selected fields per each coverslip for each condition from at least three independent experiments were analyzed.

### Statistical analysis

Data are expressed as means ± standard error of the means (SEM). One-way analysis of variance (ANOVA) followed by Bonferroni’s test was used for more than two statistical comparisons using GraphPad Prism 7 version 7.04.

## Results

### Lithium protects synapses from HIV-1 Tat-induced synapse loss

**Figure 1.**
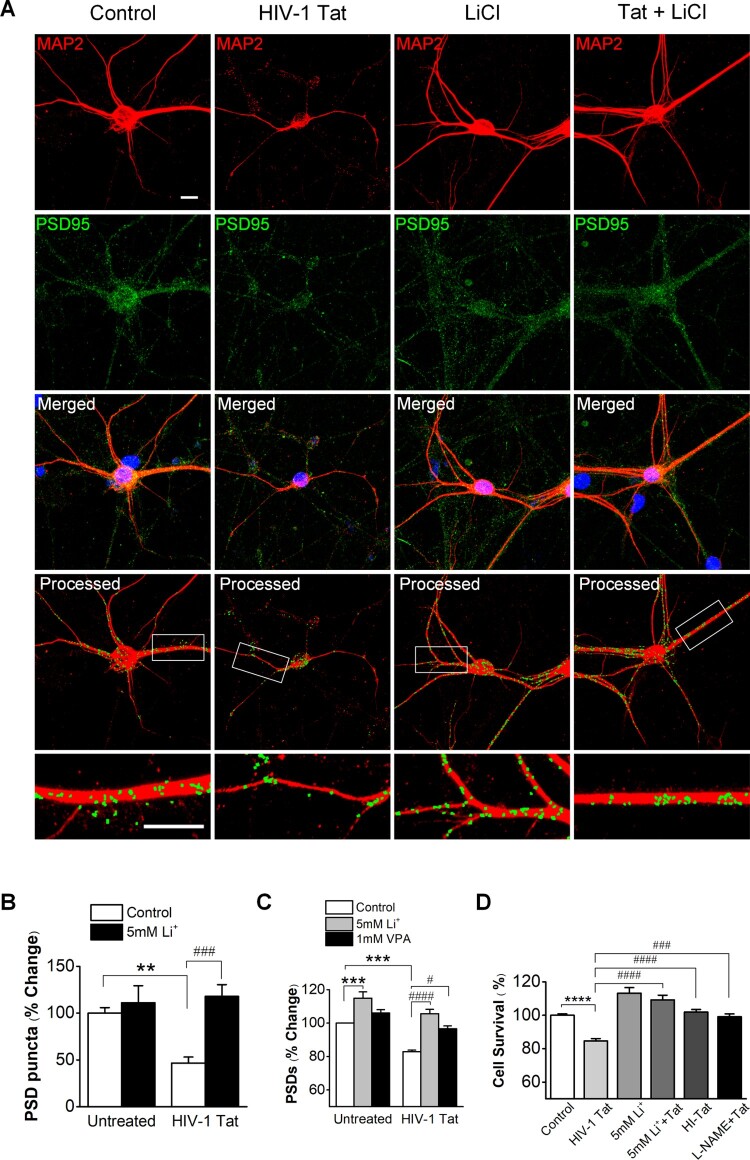
Lithium has synapto-protective effect against HIV-1 Tat-mediated synapse loss. *A*, Analyzed confocal images for synaptic puncta of hippocampal neurons expressing PSD95 and MAP2 after 24 h treatment under control (Control), 50 ng/ml HIV-1 Tat (Tat) and 5 mM Lithium-treated conditions in the presence of 50 ng/ml HIV-1 Tat (Tat + 5mM Li^+^). Analysis of PSD puncta using the algorithm of image process display lithium increased PSD puncta against HIV-1 Tat-induced synapse loss. Processed confocal images expressing PSD95 and MAP2, analyzed using Image J program. Scale bar, 100 µm. *B*, Bar graph shows significant change in the PSD puncta following treatment with 5 mM lithium in the absence (untreated) or the presence of 50 ng/ml HIV-1 Tat as indicated. Data are expressed as mean ± SEM; **p < 0.01 relative to untreated control; ^###^*p* < 0.001 relative to HIV-1 Tat alone (ANOVA with Bonferroni post test). *C*, Bar graph shows significant change in the intensity of PSDs following treatment with 5 mM lithium and 1 mM valproic acid in the absence (untreated) or the presence of 50 ng/ml HIV-1 Tat as indicated. Processing of PSD95 images identified PSDs as mean intensity after subtraction of background intensity (threshold: 3000). Data are expressed as mean ± SEM; ***p < 0.001 relative to untreated control; ^#^*p* < 0.05 and ^####^*p* < 0.0001 relative to HIV-1 Tat alone (ANOVA with Bonferroni post test). *D*, Bar graph shows cell survival changes after 48 h for untreated cells (Control) and cells treated with 5 mM lithium or 100 µM L-NAME in the absence and the presence of 50 ng/ml HIV-1 Tat (HI-Tat; heat-inactivated Tat). Data are expressed as mean ± SEM; ****p < 0.0001 relative to control; ^###^*p* < 0.001 and ^####^*p* < 0.0001 relative to 48 h HIV-1 Tat alone (ANOVA with Bonferroni post test).

HIV-1 Tat-induced reversibly loss of PSD was quantified based on PSD95 and MAP2 expressed cells as previously described (Kim et al. 2008, 2021). Changes in the number of excitatory synapses that form between cultured rat hippocampal neurons were quantified using immunocytochemistry (Kim et al. 2021). Cultured rat hippocampal neurons exposed to Tat were fixed for immunocytochemistry and labeled with antibodies specific to PSD95 immunoreactivity to PSD95 (green) for display a punctated pattern and MAP2 immunoreactivity (red) filled with soma and dendrite. Treatment with 50 ng/ml HIV-1 Tat for 24 h significantly induced a decrease in PSD95 puncta by 46.6 ± 6.5% (*n* = 12) compared with the control (100 ± 5.9%, *n* = 14), consistent with results of a previous study showing Tat-induced loss of PSD puncta (Kim et al. 2008) (Figure 1A, B).

We next determined whether lithium protects synapses from HIV-1 Tat-induced loss. Co-treatment with HIV-1 Tat and lithium significantly increased PSD95 puncta (117.9 ± 12.6%, *n* = 12) compared with the HIV-1 Tat-treated group (Figure 1A, B). These results indicate that lithium can prevent HIV-1 Tat-induced synapse loss. Treatment with HIV-1 Tat decreased green fluorescence intensity by 82.9 ± 0.9% (*n* = 15) of control (Figure 1C). HIV-1 Tat induced PSD intensity loss was significantly different from untreated and those treated with heat-inactivated Tat (98.6 ± 1.1%, *n* = 12), consistent with results of a previous study showing Tat-induced loss of PSD puncta (Kim et al. 2008). Co-treatment with HIV-1 Tat and lithium increased the PSD95 fluorescence intensity by 105.6 ± 2.6% (*n* = 15) relative to treatment with only HIV-1 Tat (82.9 ± 0.9%, *n* = 15) (Figure 1C). We also investigated whether the synapto-protective effect of lithium is reproduced by other mood stabilizer, valproate. The therapeutic concentration of valproate is as high as 0.7 mM (FI 2006). Co-treatment with valproic acid and HIV-1 Tat preserved the PSD95 intensity (96.7 ± 1.6%, *n* = 9) relative to control at a therapeutically relevant concentration of 1 mM, though not as great as lithium (Figure 1C). This result suggests that the protection provided by lithium from the synaptic damage induced by HIV-1 Tat has more reinforced effects than other mood stabilization.

To further examine the effect of lithium on Tat-induced neuronal death, cultured rat hippocampal neurons were cotreated with Tat and lithium. Cell viability was measured using MTT assay at 48 h after exposure. As shown in Figure 1D, Cell viability at 48 h after exposure to Tat was 84.6 ± 1.5% (*n* = 12), which was decreased compared to that of untreated control (100 ± 0.9%, *n* = 12). However, cell viability at 24 h was 99.2 ± 3.3% (*n* = 12, data not shown), which was not decreased compared to that of untreated control. Such significant decrease in cell viability at 48 h after exposure to Tat indicates that Tat-induced synapse loss precedes overt neuronal death. In the group co-treated with HIV-1 Tat and lithium, lithium significantly inhibited neuronal death caused by Tat at 48 h post exposure with cell viability of 109.2 ± 2.8% (*n* = 12). The significant neuroprotective effect of lithium found in this study was consistent with the result of a previous study (Maggirwar et al. 1999).

### The synapto-protective effect of lithium against HIV-1 Tat-induced synapse loss is mediated by the activated NMDA receptors

**Figure 2.**
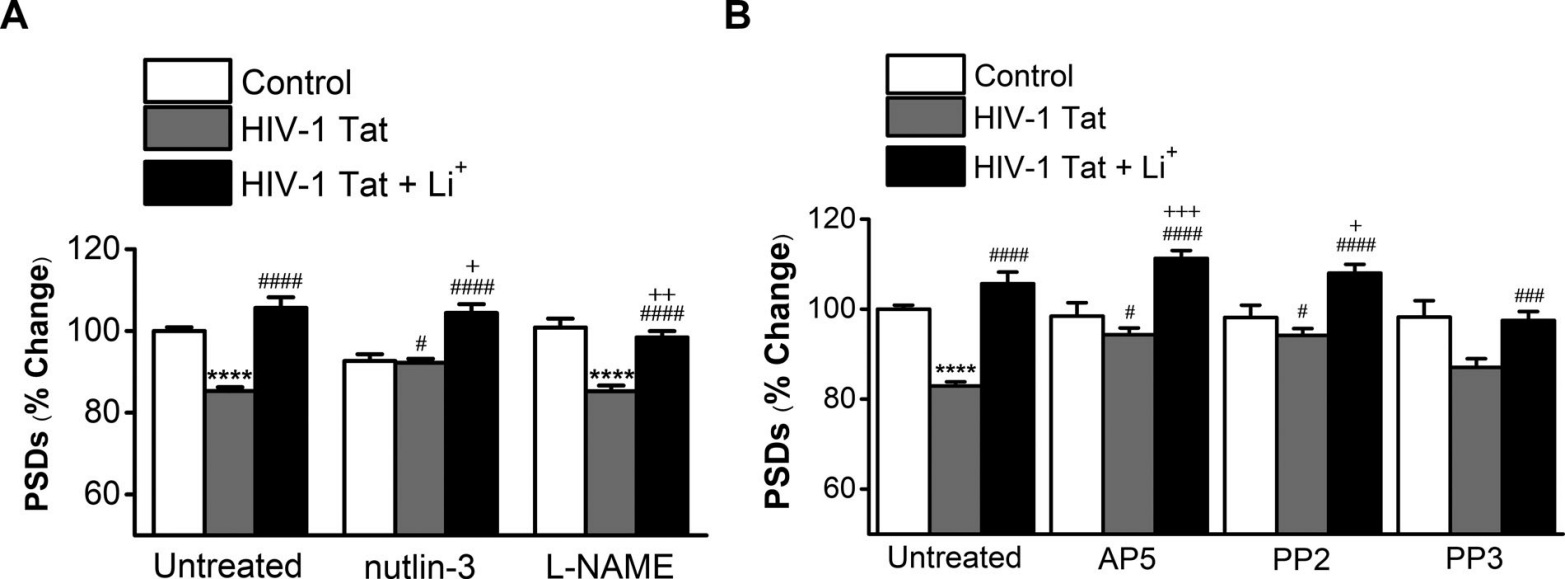
Lithium potentiates synapto-protective effect mediated by activation of NMDA receptors. *A*, Bar graph summarizes the effects of inhibitors on changes in PSD95 intensity (PSDs) 24 h after treatment under control, HIV-1 Tat-treated (HIV-1 Tat) or HIV-1 Tat and Lithium-treated conditions (HIV-1 Tat + Li^+^). Cultures were treated with 1 µM Nutlin-3 or 100 µM L-NAME for 30 min following addition of HIV-1 Tat. Data are expressed as mean ± SEM; ****p < 0.0001 relative to untreated control; ^#^*p *< 0.05 and ^####^*p *< 0.0001 relative to HIV-1 Tat alone (Untreated); ^+^*p *< 0.05 and ^++^*p *< 0.01 relative to HIV-1 Tat + inhibitors (ANOVA with Bonferroni post test). *B*, Bar graph summarizes the effects of inhibitors associated with activation of NMDA receptor on changes in PSD95 intensity (PSDs) 24 hr after treatment under control, HIV-1 Tat-treated (HIV-1 Tat) or HIV-1 Tat and Lithium-treated conditions (HIV-1 Tat + Li^+^). Cultures were treated with 20 µM AP5 for 15 min, 10 µM PP2 and 10 µM PP3 for 1 h following addition of HIV-1 Tat. Data are expressed as mean ± SEM; ****p < 0.0001 relative to untreated control; ^#^*p *< 0.05, ^###^*p *< 0.001 and ^####^*p *< 0.0001 relative to HIV-1 Tat alone (Untreated); ^+^*p *< 0.05 and ^+++^*p *< 0.001 relative to HIV-1 Tat + inhibitors (ANOVA with Bonferroni post test).

Activation of NMDA receptors induces PSD loss through ubiquitin-proteasome pathway (Colledge et al. 2003; Waataja et al. 2008). Similarly, HIV-1 Tat induces PSD loss via the ubiquitin-proteasome pathway (Kim et al. 2008). We investigated the mechanism by which lithium could provide synapto-protective effect on HIV-1 Tat-induced synapse loss. Pretreatment with 1 µM Nutlin-3, inhibitor of ubiquitin E3 ligase MDM2 (murine double minute 2), for 30 min abolished HIV-1 Tat induced loss of PSD95 intensity (Figure 2A), indicating that HIV-1 Tat-induced synapse loss is dependent on the ubiquitin-proteasome pathway. In treatment with HIV-1 Tat and lithium, Nutlin-3 still showed synapto-protective effect by lithium, but did not show any additional protection. Application of L-NAME (100 µM) did not have any significant effect in the HIV-1 Tat-induced synapse loss or on the synapto-protective effect of lithium against HIV-1 Tat-induced synapse loss.

Specifically, we examined whether lithium could affect HIV-1 Tat-induced synapse loss via the activation of NMDA receptor initiated by cellular uptake of Tat. Pretreatment with 20 µM AP5, NMDA receptor inhibitor, for 15 min significantly inhibited HIV-1 Tat-induced synapse loss (Figure 2B) (94.3 ± 1.5%, *n* = 6). Treatment with AP5 potentiated PSD intensity in the HIV-1 Tat and lithium co-treated neurons (111.3 ± 1.7%, *n* = 6). Furthermore, pretreatment with Src kinase inhibitor PP2 (10 µM) for 1 h further potentiated the synapto-protective effect of lithium on synaptic damage by HIV-1 Tat (PP2 + Tat + Li^+^; 108 ± 2.0%, *n* = 6 vs. PP2 + Tat; 92.9 ± 1.6%, *n* = 6, *p* = 0.0108). PP2 also blocked HIV-1 Tat-induced synapse loss (PP2 + Tat vs. HIV-1 Tat only; 82.9 ± 0.9%, *n* = 15, *p* = 0.0162) as shown in the previous results (Kim et al. 2008). Whereas pretreatment with PP3, the inactive form of PP2, did not significantly affects PSDs changes in the HIV-1 Tat and lithium co-treated neurons (97.5 ± 2.0%, *n* = 6). These data suggest that lithium affects HIV-1 Tat-induced synapse loss via the activated NMDA receptor.

### Both inhibition of inositol depletion and GSK-3*β* inhibition mediate the synapto-protective effect of lithium against HIV-1 Tat-induced synapse loss

**Figure 3.**
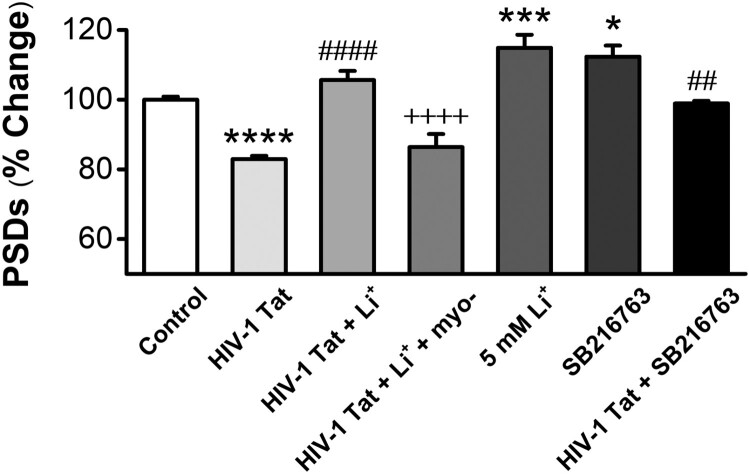
GSK-3*β* inhibitor and inositol depletion increases the intensity of PSDs against HIV-1 Tat-induced synapse loss. Bar graph summarizes the effects of GSK-3*β* inhibitor SB216763 and inositol depletion on changes in the intensity of PSD95. Data are expressed as mean ± SEM; *p < 0.05, ***p < 0.001 and ****p < 0.0001 relative to control; ^##^*p* < 0.05 and ^####^*p* < 0.0001 relative to HIV-1 Tat alone; ^++++^*p* < 0.0001 relative to HIV-1 Tat + Li^+^ (ANOVA with Bonferroni post test).

Lithium’s mechanism of action has been associated with both an inhibition of GSK-3β and inositol depletion (Berridge 1989; Wexler et al. 2008). Previous studies have shown that the inhibition of GSK-3β has the proliferative and anti-apoptotic effects (Wexler et al. 2008), and inositol depletion mediates the effects of lithium on synapse formation (Kim and Thayer 2009). We investigated which mechanism of lithium mediates the synapto-protective effect of lithium on the synapse loss by HIV-1 Tat. Inhibition of inositol phosphatases results in the free inositol depletion for phosphoinositide-mediated intracellular signaling cascades (Berridge 1989). If inhibition of inositol phosphatases is the site for synapto-protective effects of lithium against HIV-1 Tat-induced synapse loss, then providing exogenous myo-inositol should block the effects of lithium on PSD95 immunoreaction fluorescent intensity. Myo-inositol (1 mM) inhibited the synapse protection afforded by lithium (Tat + Li^+^; 105.6 ± 2.6, *n* = 15 vs. Tat + Li^+^ + myo-; 86.4 ± 3.7%, *n* = 9, *p* < 0.0001) (Figure 3).

If GSK-3β is a major molecular target for the action of lithium to protect synapses against HIV-1 Tat-induced synapse loss, inhibition of GSK-3β by other drugs should mimic lithium-induced synapto-protective effect on synapse loss by Tat. Pretreatment with the GSK-3β inhibitor SB216763 significantly inhibited HIV-1 Tat-induced synapse loss (98.9 ± 0.7%, *n* = 9) (Figure 3). Application of only SB216763 also induced the increase of PSDs on normal neurons (112.3 ± 3.2%, *n* = 9). Thus, both inositol depletion and inhibition of GSK-3β mediates the synapto-protective effect of lithium on synapse loss by HIV-1 Tat.

## Discussion

**Figure 4.**
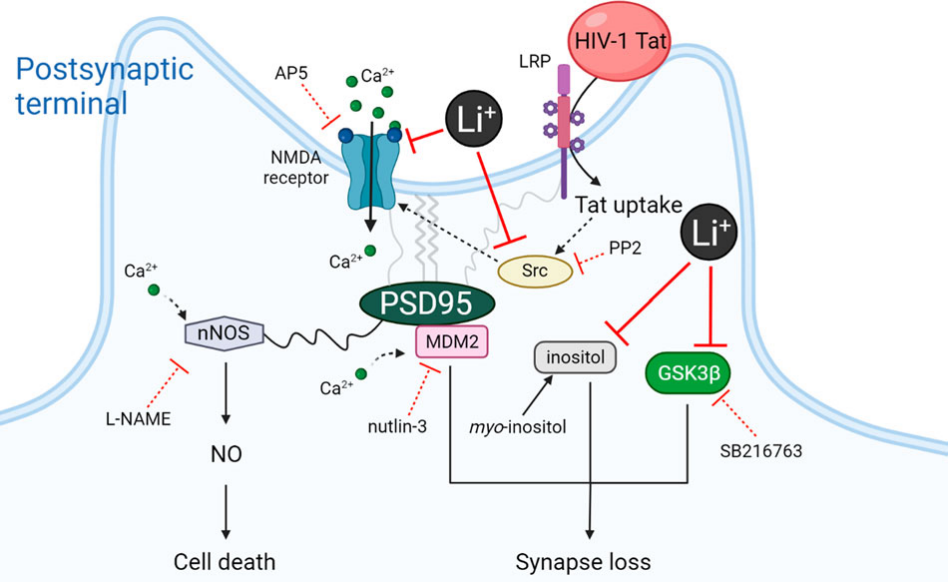
Schematic diagram illustrating the proposed mechanism of synapto-protective effects induced by lithium.

In the present study, we investigated whether the mood stabilizer lithium could exert a protective effect on synaptic damage induced by HIV-1 Tat protein, a pathological model of HAND. Our results showed that lithium not only inhibited synaptic damage by Tat, but also protected neurons against Tat-induced neurotoxicity. The synapto-protective effect of lithium against Tat-induced neurotoxicity was also mediated by the activated NMDA receptor, and mediated by both inhibition of GSK3β and inositol depletion, known as the main mechanisms of action of lithium as described in the schematic diagram (Figure 4).

The reported neuroprotection by lithium suggests it could be used as a therapeutic agent to prevent synapse loss that underlies cognitive impairment induced by HIV-1 and to treat symptoms of mood disorder in HAND. The therapeutic concentration of extracellular lithium of bipolar patients is in the range of 0.8 to 1.2 mM, and thus the synaptic effects of lithium occurred at clinically relevant concentrations (FI 2006). The synaptogenic effect of lithium has been reported to be much faster than the therapeutic effects of lithium, which requires several weeks (Kim and Thayer 2009). In the *in vitro* model used here, lithium-induced synapse formation was shown at 1 mM but did not reach significant levels (102.3 ± 1.6%, *n* = 6) (Supplementary Figure 1). However, significant synapse formation was observed at concentrations above 5 mM (114.9 ± 3.7%, *n* = 15 at 5 mM; 142.5 ± 9.2%, *n* = 6 at 10 mM) (Supplementary Figure 1). The increase was significant when the concentration of lithium was 5 mM or more. This tendency was consistent with results of a previous study showing lithium-induced synapse formation. Such synapse formation may act as an early event contributing to mood stabilization.

Here, we used changes in the intensity of PSD95 immunoreactive fluorescence to quantify synaptic changes between cultured rat hippocampal neurons described previously (Jaafari et al. 2013). The experimental results obtained by this method were consistent with the previous results that Tat induced loss of PSD95 puncta (Kim et al. 2008). Furthermore, synapse loss by Tat is known to be mediated by the ubiquitin-proteasome pathway (Kim et al. 2008). We also confirmed this by the action of inhibitors involved in the ubiqutin-proteasome pathway, and these results, derived from our experimental condition, were consistent with previous reports (Kim et al. 2008).

Lithium is a drug widely used as a mood stabilizer for bipolar disorder for decades (Licht 2012). Lithium has been reported to reduce the severity of neurodegenerative disorders such as Alzheimer’s disease (AD). Moreover, there is much evidence that lithium may be effective in an array of other common CNS disorders, including stroke, Parkinson’s disease, and Huntington’s disease (Young 2009; Inestrosa and Varela-Nallar 2014). Lithium has previously been reported to reduce neuronal cell death in several neurodegenerative diseases, including Parkinson’s diseases (Youdim and Arraf 2004; Wada et al. 2005; Chiu et al. 2013), amyotrophic lateral sclerosis (De Sarno et al. 2008) and multiple sclerosis (Watase et al. 2007). Though the neuroprotective effect of lithium was found for years, the effect of lithium on synapse dysfunction induced by HIV-1 Tat protein, which can reproduce HAND, is not yet known. In our present study, lithium prevented Tat-induced synapse loss and subsequent Tat-induced neurotoxicity.

HIV-1 Tat protein plays a prominent role by activation of NMDA receptors and subsequent activation of down-stream signaling pathway, which results in impairment of cognitive function (Haughey et al. 2001; Song et al. 2003; Fitting et al. 2010; Bachani et al. 2013; Krogh et al. 2015). HIV-1 Tat, for the first step, binds to the LRP and activates NMDA receptors to induce Ca^2+^ influx, which ultimately contributes to synaptic dysfunction and subsequent neurotoxicity. AP5 and PP2 not only prevented the decrease of PSD intensity induced by Tat, but also potentiated the synapto-protective effect of lithium. These data suggest that the synapto-protective effect of lithium may be induced by activation of NMDA receptors as an initial step. In fact, lithium has been shown to reduce tyrosine phosphorylation of proline-rich tyrosine kinase 2 (Pyk2), which leads to the inhibition of the activation of Src, leading to a reduction of tyrosine kinase-mediated NMDA receptor subunits phosphrylation by lithium (Hashimoto et al. 2002; Ma and Zhang 2003).

In addition, we found that lithium exerts a synapto-protective effect mediated by the inositol depletion through the result showing that exogenously administered myo-inositol prevented Tat-induced synaptic damage. However, lithium is known to have neuroprotective effects in various models through the inhibition of GSK-3β. In fact, in our results, SB216763, an inhibitor of GSK-3β, also mimicked the synapto-protective effect of lithium on HIV-1 Tat-induced loss of synapse. Taken together, these data suggest that the typical mechanism of action of lithium might be also a mechanism to explain the action of lithium to synapto-protect the synaptic damage by HIV-1 Tat. Moreover, valproic acid, another mood stabilizer, increased the PSD intensity at a clinically relevant concentration against Tat-induced decreases of PSD intensity. Previous studies have shown that valproic acid is clinically effective in HIV-associated cognitive impairment (Schifitto et al. 2006).

In conclusion, we showed that a synapto-protective effect of lithium on synapse loss and neuronal death triggered by HIV-Tat. We also suggested the underlying mechanism of action of lithium, including NMDA activation, inositol depletion and GSK3β inhibition, which protect synaptic damage by HIV-1 Tat. These findings suggest lithium can be useful as an effective therapeutic agent to HIV-induced neurodegenerative disorders including HAND.

## Supplementary Material

Supplemental Material
