# Supplementary figures and images for: Synapto-protective effect of lithium on HIV-1 Tat-induced synapse loss in rat hippocampal cultures
Source: Anim Cells Syst (Seoul). 2021 Dec 27;26(1):1–9. doi: 10.1080/19768354.2021.2018044 (PMC8928815; doi:10.1080/19768354.2021.2018044)

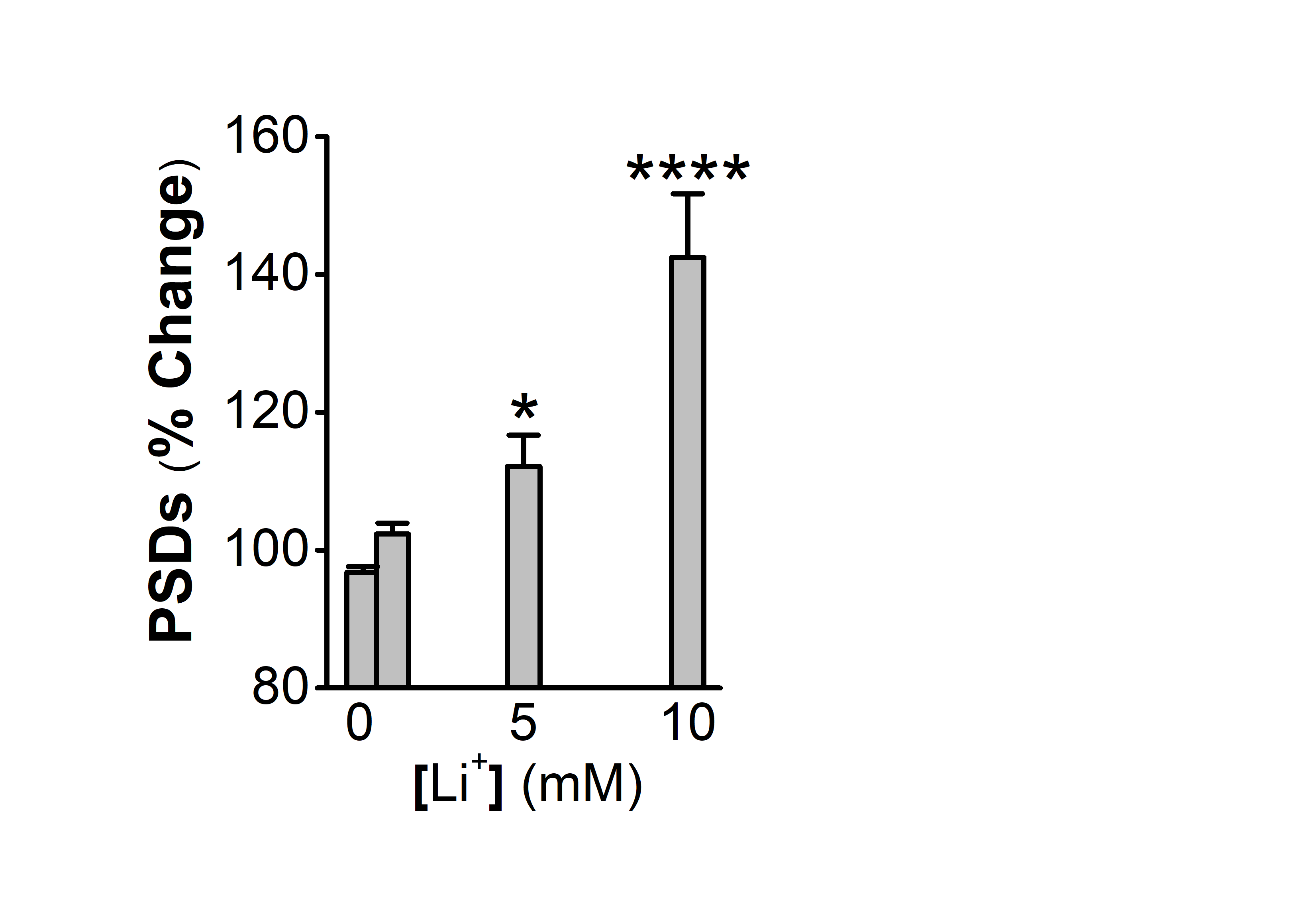

Supplement: Supplemental Material [file TACS_A_2018044_SM1787.tif]
